# Executive Functions, Anxiety, Social Participation and Quality of Life in Children with Migraine During COVID-19

**DOI:** 10.3390/life15040528

**Published:** 2025-03-23

**Authors:** Jacob Genizi, Hila Samet, Hussein Zaitoon, Uriel Elimelech, Nogah C. Kerem, Aharon Kessel, Adel Shalata, Keren Nathan, Batya Engel-Yeger

**Affiliations:** 1 Pediatric Department, Bnai Zion Medical Center, Haifa 3104802, Israel; hussein.zaitoon@gmail.com (H.Z.); urielelimelech@gmail.com (U.E.); nogah.kerem@b-zion.org.il (N.C.K.); keren.nathan@b-zion.org.il (K.N.); 2 Rappaport Faculty of Medicine, Technion Israel Institute of Technology, Haifa 3109601, Israel; aharon.kessel@b-zion.org.il (A.K.); adel.shalata@b-zion.org.il (A.S.); 3 Occupational Therapy Department, University of Haifa, Haifa 3104802, Israel; hilasamet@gmail.com (H.S.); bengel@univ.haifa.ac.il (B.E.-Y.); 4 Adolescent Medicine Unit, Bnai Zion Medical Center, Haifa 3104802, Israel; 5 Division of Allergy and Clinical Immunology, Bnai Zion Medical Center, Haifa 3104802, Israel; 6 Simon Winter Institute for Human Genetics, Bnai Zion Medical Center, Haifa 3104802, Israel

**Keywords:** executive functions, anxiety, participation, quality of life, children, headache, migraine

## Abstract

Objective: We aimed to compare executive functions (EF), anxiety, social participation, and quality of life (QoL) between children with migraine and healthy controls during the COVID-19 pandemic, and to examine these parameters in children in each group who did vs. did not contract COVID-19. Materials and Methods: A prospective cohort study was carried out. The patient group comprised children seen in our pediatric neurology clinic for migraine, and the control group was composed of aged-matched healthy children with no neurological findings or developmental disorders. The participants’ parents completed a health and demographic questionnaire, the BRIEF (child/adolescent version), the PedsQL, the State–Trait Anxiety Inventory for Children (STAIC), and the CASP. The participants or their parents furnished information on whether the participant had contracted COVID-19. Results: A total of 84 children and adolescents aged 6–17.5 (mean of 12.8) participated in the study, including 33 with migraine (17 boys, 16 girls) and 51 healthy controls (28 boys, 23 girls). The children with migraine showed significantly lower EF due to reduced behavioral regulation, higher trait anxiety, and lower physical, emotional, and school-related QoL. Reduced EF correlated with the intensity of migraine attacks, higher anxiety, reduced social participation, and reduced QoL. Lower social participation correlated with reduced QoL and predicted emotional and social QoL. The BRIEF metacognition scale predicted school-related QoL. Healthy children who contracted COVID-19 showed significantly lower EF than children with migraine in the inhibition (56.66 ± 10.56 vs. 45.71 ± 7.12, *p* = 0.013) and initiation (60.01 ± 11.89 vs. 46.01 ± 6.54, *p* = 0.005) BRIEF scales, and in the general metacognition index (65.83 ± 14.48 vs. 46.75 ± 9.19, *p* = 0.003). Healthy children who contracted COVID-19 had significantly worse initiation and working memory compared to those who did not contract COVID-19 (initiation: 60.01 ± 11.89 vs. 46.81 ± 8.89, *p* = 0.007), working memory: 61.16 ± 15.48 vs. 47.21 ± 11.06, *p* = 0.021). Conclusion: Migraine has a significant negative impact on executive functions in children and adolescents, greater than contracting COVID-19. Executive dysfunction influences patients’ emotional state, participation in social activities, and quality of life. The COVID-19 pandemic had a less deleterious effect on migraine patients compared to the healthy control group. Further research on pediatric migraine is warranted.

## 1. Introduction

Headaches are common in children and adolescents, and the most frequently reported pain complaint among those seeking medical attention [1]. Migraine is one of the most prevalent headache disorders in this population, especially among adolescents: the prevalence of migraine rises from 3% during the preschool years to 23% among high-schoolers [2]. Changes in sleep patterns, dietary habits, physical activity, and emotional stress all contribute to the worsening of migraine in children [3,4].

The COVID-19 pandemic profoundly affected the wellbeing of children and adolescents, impacting various aspects of their physical and mental health. Disrupted routines, prolonged periods of social isolation, increased screen time, and heightened stress and anxiety related to the pandemic raised concerns about a potential amplification in the frequency and severity of childhood migraines. Indeed, numerous studies have documented an upsurge in migraine symptoms and frequency among children during the pandemic [5,6,7]. Special attention was given to dizziness, which was exacerbated during the acute infection and mandatory quarantine periods [8]. Additionally, restricted access to healthcare services and disrupted follow-up appointments during the pandemic added challenges to the management and treatment of childhood migraine [9,10].

Our study aimed to explore the impact of the COVID-19 pandemic on the executive functions (EF) and quality of life (QoL) of children with migraine and healthy controls, and to examine the impact—if any—of contracting COVID-19. We compared our outcome variables between children with migraine and healthy controls, and then divided both groups based on whether the child contracted COVID-19 during the pandemic. We conducted within- and between-group comparisons with respect to EF, anxiety, social participation, and QoL, and examined correlations among these variables. Based on the correlation results, we further examined whether EF, anxiety, participation, and migraine severity predicted QoL.

Our main hypotheses were as follows: (1) The children with migraine would have significantly lower EF, higher anxiety scores, lower social participation, and lower QoL than the healthy controls. (2) Of the four groups (children with migraine vs. healthy controls and the children who did vs. did not contract COVID-19), the children with migraine who were affected by COVID-19 would have the lowest EF, highest anxiety, lowest social participation, and lowest QoL. (3) Among the children with migraine, significant correlations would be found between their migraine severity (as measured by the PedMIDAS) and their EF, anxiety, participation, and QoL. (4) Migraine severity, EF, anxiety, and participation would significantly predict QoL.

## 2. Materials and Methods

### 2.1. Participants and Procedure

Out of the 100 children recruited, 84 children between the ages of 6 and 17.5 years (mean age of 12.8 ± 2.75) completed this study. The study group included 33 children with episodic migraine (17 boys, 16 girls) who were prospectively recruited from the outpatient pediatric neurology clinic at the Bnai Zion Medical Center during the years 2020–2022. The control group included 51 healthy children (28 boys, 23 girls) with no significant illness, no developmental disorders, and no neurological findings.

Children from the study group were recruited during their visit to the neurology clinic. All children in the study group met the diagnostic criteria for migraine according to the International Classification of Headache Disorders, 3rd edition (ICHD-3) [11]. The patients and their parents/caregivers were asked to complete the questionnaires during their clinic visit (after providing informed consent).

The children in the control group were recruited through advertisements. The parents of 70 children responded, of whom 51 met the inclusion criteria (children aged 6–17 with no significant illness, no developmental disorders, and no neurological findings). Again, informed consent was obtained from all participants. The controls were evaluated in their homes by a member of the research team.

This study received ethics approval from the Bnai Zion Medical Center Ethics Review Board (BNZ 185-20) and was conducted in accordance with the Declaration of Helsinki. The demographic details of the study and control group participants are summarized in Table 1.

### 2.2. Materials

All patients in the study group underwent a physical and neurological assessment by a pediatric neurologist and provided a thorough headache history. The patients (or their parents) also filled in the PedMIDAS questionnaire—a tool developed specifically to assess migraine disability in pediatric and adolescent patients during prospective data collection, which has been tested and validated for ages 4 to 18 [12]. All participants (both migraine patients and controls) answered a set of general health questions, including whether or not the participant contracted COVID-19 during the pandemic. We defined contracting COVID-19 as actually developing symptoms, not merely testing positive for the presence of the SARS-CoV-2 virus.

For the main measures, all participants filled in the following questionnaires:

1. *Behavior Rating Inventory of Executive Functions (BRIEF)*: The BRIEF is a behavioral rating measure for children and youth aged 5–18 years, which aims to measure EF as expressed in daily life situations (for example: “becomes upset with new situations”; “has a messy desk”; “is disturbed by change of teacher or class”; “does not check work for mistakes”; “has trouble concentrating on chores, schoolwork”). The BRIEF includes 86 items summarized into two indexes: the behavioral regulation index (BRI), which includes inhibition, shifting, and emotional control scales; and the metacognition index (MI), which includes initiation, working memory, planning, organization of materials, and monitoring scales. The BRI and MI scores are combined to generate a global executive composite (GEC) score. In the present study, we used the parents’ BRIEF reports. Parents rated how frequently their child expressed the behavior described in each item on a Likert-type scale ranging from 1 (infrequently) to 3 (often), such that higher scores indicate lower EF. All raw scores were converted to standard scores. A standard GEC score of 65 indicates deficiencies in executive functions. The BRIEF is considered to have good psychometric properties [13,14,15].
2. *State–Trait Anxiety Inventory for Children (STAIC)* [16]: The STAIC comprises two indexes: the STAIC T-Anxiety scale, designed to measure a general proneness to anxious behavior rooted in the personality, and the STAIC S-Anxiety scale, which measures anxiety as a fleeting emotional state. The two indexes each include twenty statements that ask, respectively, how the child generally feels (trait anxiety), and how the child feels at a particular moment in time (state anxiety). Higher mean scores represent lower anxiety.
3. *Child and Adolescent Scale of Participation (CASP)* [17]: This scale measures the extent to which children participate in home, school, and community activities compared to children of the same age as reported by family caregivers. The CASP was developed as part of the Child and Family Follow-up Survey (CFFS) to monitor outcomes and needs of children with traumatic and other acquired brain injuries [17]. The content and methods used in the CASP and CFFS were informed by the International Classification of Functioning, along with other research addressing the social participation of children and youth with a range of disabilities. The CASP consists of 20 ordinal-scaled items in four subsections: home participation (6 items), community participation (4 items), school participation (5 items), and home and community living activities (5 items) (sample items can be found in the Results section, under Correlations). All items are rated on a four-point scale: “age expected (full participation)”, “somewhat restricted”, “very restricted”, and “unable” (with a “not applicable” option for activities in which the child would not be expected to participate due to age, such as work). Most items are applicable to children aged five and older. Higher scores reflect greater age-expected participation. The CASP has reported evidence of test–retest reliability (intraclass correlation coefficient = 0.94), internal consistency (α ≥ 0.96), and construct and discriminant validity.
4. *Pediatric Quality of Life Inventory (PedsQL)* [18]: We used Version 4.0 of the child’s report, which profiles children’s health-related quality of life (HRQoL) in four dimensions: physical functioning (eight items), emotional functioning (five items), social functioning (five items), and school functioning (five items). Emotional and social functioning are considered together in a higher-order dimension of psychosocial health. The child indicates the frequency of problems during the past month on a five-point Likert scale (0 = never a problem; 1 = almost never a problem; 2 = sometimes a problem; 3 = often a problem; 4 = almost always a problem). Items are then transformed into a 0–100-point scale (0 = 100; 1 = 75; 2 = 50; 3 = 25; 4 = 0) to produce the HRQoL percentage. A higher percentage indicates a better HRQoL.

### 2.3. Data Analysis

The data were analyzed using SPSS-25 software. Descriptive statistics were calculated for all measures. Normality tests revealed normal distribution for most scales. Chi square analysis was used to test for significant differences between the groups in relevant socio-demographic parameters, followed by z-tests where required. *t*-tests were used to test for differences between the groups in age and in total scores, while MANOVA was used to test for differences in the various subscales. Correlations among the measures were examined using Pearson’s correlation test. Finally, stepwise linear regression was performed to examine the relative contribution of migraine severity, EF, anxiety, and social participation to each of the QoL domains. Significance levels were adjusted to account for multiple testing for all analyses.

## 3. Results

In order to eliminate the influence of headaches, we first report the results between the two main groups (children with migraine vs. healthy controls) for each of the main variables, without regard to whether or not they had contracted COVID-19. We then report comparisons between the relevant subgroups.

### 3.1. Executive Functions

The majority of children from both main groups (children with migraine and healthy controls) had normal BRIEF scores (86.2% of the former and 84.6% of the latter). Hence, no significant differences were found between the two main groups in the total BRIEF scores. However, when comparing the mean scores of the BRIEF subscales, the healthy controls showed slightly lower initiation than the children with migraine (see Table 2).

### 3.2. Executive Functions in Children with Migraine vs. Healthy Controls (Participants Who Did Not Contract COVID-19)

In children who did not contract COVID-19, emotional control was significantly worse in the children with migraine (57.77 ± 11.97) compared to the healthy controls (45.72 ± 9.46; F_1,53_ = 6.42, *p* = 0.014). The children with migraine also scored significantly worse on the organization of materials scale (52.51 ± 11.69) compared to the healthy controls (46.71 ± 11.88; F_1,53_ = 3.98, *p* = 0.05).

### 3.3. Executive Functions in Children with Migraine vs. Healthy Controls (Participants Who Contracted COVID-19)

Here, an opposite trend was found. Among the children who contracted COVID-19, the healthy controls scored significantly worse than the children with migraine in two of the BRIEF scales: inhibition (56.66 ± 10.56 vs. 45.71 ± 7.12 for the controls and migraine group, respectively; F_1,53_ = 7.81, *p* = 0.013) and initiation (60.01 ± 11.89 vs. 46.01 ± 6.54, respectively; F_1,53_ = 10.76, *p* = 0.005). In addition, the healthy children scored worse in the overall metacognition index (65.83 ± 14.48 vs. 46.75 ± 9.19, respectively; F_1,53_ = 11.77, *p* = 0.003).

### 3.4. Executive Functions in Healthy Children Who Did vs. Did Not Contract COVID-19

The healthy children who contracted COVID-19 had significantly worse initiation and working memory compared to those who did not contract the disease (initiation: 60.01 ± 11.89 vs. 46.81 ± 8.89, F = 8.74, *p* = 0.007; working memory: 61.16 ± 15.48 vs. 47.21 ± 11.06, F = 6.13, *p* = 0.021).

### 3.5. Executive Functions in Children with Migraine Who Did vs. Did Not Contract COVID-19

Among the children with migraine, the only significant difference between those who did and did not contract COVID-19 was found in monitoring. Monitoring was worse in children who did not contract COVID-19 (53.47 ± 10.48 vs. 43.41 ± 6.64; F = 8.56, *p* = 0.007).

### 3.6. Anxiety

Looking only at the two main groups (i.e., ignoring COVID-19 status), the children with migraine had significantly higher state anxiety than the healthy controls.

We next compared anxiety within each of the two main groups by COVID-19 status. Among the healthy children, we found differences between those who did and those who did not contract COVID-19 in both anxiety measures. Specifically, the COVID–yes group had significantly lower state anxiety compared to the COVID–no group (mean state = 1.86, SD = 0.33 vs. mean state = 2.34, SD = 0.27, respectively, F_1,30_ = 18.64, *p* < 0.001), but higher trait anxiety (mean trait = 2.11, SD = 0.62 vs. mean trait = 1.34, SD = 0.38, respectively). By contrast, among the children with migraine, there were no significant differences between the COVID subgroups in either state anxiety (mean = 2.41, SD = 0.48 vs. mean = 2.26, SD = 0.31) or trait anxiety (mean = 1.69, SD = 0.38 vs. mean = 1.74, SD = 0.43).

### 3.7. Social Participation

No significant differences were found between the children with migraine and the healthy controls. In addition, no significant impact of contracting COVID-19 was found in either group: the children did not differ based on their COVID status in any of the participation scales.

### 3.8. Quality of Life

The children with migraine had a lower QoL than the healthy controls in all measured domains. Significant differences between the groups were found in physical, emotional, and school-related QoL (see Table 3).

No significant interaction was found between the main group and COVID-19 status. Within both the migraine and control groups, the children who did vs. did not contract COVID-19 did not differ in any of the QoL domains.

## 4. Discussion

This study aimed to compare executive functions, anxiety, social participation, and quality of life between children with migraine and healthy controls; to explore the relationships among the examined factors in children with migraine; and to examine the implications of contracting COVID-19 for the examined factors. We hypothesized that (1) the children with migraine would have significantly lower EF, higher anxiety, lower participation, and lower QoL than the healthy controls; (2) the children with migraine who contracted COVID-19 would have the lowest EF, highest anxiety, lowest participation, and lowest QoL of the four groups; (3) among the children with migraine, significant correlations would be found between migraine severity and the four main variables; and (4) migraine severity, EF, anxiety, and participation would significantly predict QoL.

### 4.1. Executive Functions in Pediatric Migraine

Our first hypothesis was largely supported. Looking only at the presence or absence of migraine and ignoring COVID-19 status, we found that migraine patients had worse executive functions, particularly in the emotional control and organization of materials scales. We also found that EF, as measured by the BRIEF scale correlated with quality of life.

Previous research has shown that individuals suffering from migraine may experience difficulties in various cognitive domains and executive functions. These studies have found challenges with selective and divided attention, working memory, short- and long-term verbal memory, information processing speed, cognitive flexibility and inhibitory control in individuals with migraine compared to healthy individuals [4,19,20,21,22,23,24]. Several mechanisms have been postulated to account for such executive function deficits in individuals with migraine. One theory proposes that disruptions in brain networks and changes in cerebral blood flow during migraine attacks could contribute to the impairment of executive functioning. The coexistence of migraine with other conditions known to impact executive functions, like anxiety and depression, may exacerbate these deficits even further [22]. There is also evidence that individuals with migraine may still exhibit cognitive impairments even during headache-free intervals, including executive function deficits. This suggests that the cognitive effects of migraine are not solely attributable to acute headache symptoms but may involve underlying neurobiological and neurochemical mechanisms [25].

Importantly, cognitive and EF impairments observed in individuals with migraine can have substantial consequences on their daily functioning, academic achievement, and overall quality of life, especially among children and adolescents. However, most studies about EF in children with migraine have used neuropsychological tests [4,19,20,21]. Our study used the BRIEF—an ecologically valid evaluation that reflects how EF is expressed in daily life, and therefore, conveys information about the implications of EF deficits outside the clinic setting. Understanding these real-life challenges may support the development of improved interventions aimed at meeting the child’s and family’s specific needs, and so improve both daily functioning and wellbeing [26].

### 4.2. Pediatric Migraine, Anxiety, and Social Participation

In the current research, the most significant difference between the children with migraine and the healthy children was in their emotional regulation. In this area, the BRIEF questionnaire includes questions related to exaggerated emotional responses, frequent mood changes, and irritability. Migraine is a disease associated with anxiety and emotional difficulties. Many studies have found stress to be the most common trigger for headaches, particularly stress related to school [27,28]. These studies may explain the clear connection found between migraines and emotional regulation. Difficulty in emotional regulation contributes to difficulties in behavioral control, and may affect the functioning and quality of life of children with migraines [29]. Therefore, emotional regulation is a central target for intervention.

In our study, children with migraine had significantly higher state anxiety than healthy controls, although no significant differences were found in the social participation between the children with migraine and the healthy controls. This may be due to the relatively small sample size, as well as the unique effects of the COVID-19 pandemic, which restricted social interaction. Given that social participation is a critical factor in children’s development and wellbeing, future studies should examine this question in larger samples.

### 4.3. Pediatric Migraine and Quality of Life

The other main outcome of the present study was that the children with migraine had a lower quality of life in various domains as compared to the healthy controls. This finding is supported by previous reports. For example, Powers [3,30] found that migraine may reduce children’s QoL, and that this impact may differ by age group. Teens reported lower school functioning than older and younger children, and younger children reported lower social functioning than older children and teens. Physical complaints, as well as mental problems, can adversely affect a patient’s quality of life [31]. This may be reflected directly by children’s self-reports, as found in our study. The impact on quality of life was also shown [32] among children with chronic rhinitis, where the child’s health issues were found to influence the parents’ emotions, the child’s pain and discomfort, and the child’s general perception of health. The present study is the first, to our knowledge, to find a correlation between reduced social quality of life in children with migraine and the PedMIDAS score.

With respect to the association between school-related quality of life and the severity of migraines, the current findings partially support our third and fourth hypotheses. This finding is also consistent with the existing literature. Numerous studies have found reciprocal relationships between migraines and school functioning, with school-related factors both triggering migraines and being influenced by them [27,33]. Attendance and participation in school play a significant role in children’s lives, impacting their overall development, self-confidence, and self-esteem. Absence from elementary school significantly reduces current and future academic performance [34]. Involvement in school improves students’ attitudes toward learning and their academic achievement [35], enhances students’ emotional and social skills [36], and predicts a lower risk of drug use and delinquency [37]. Therefore, it is essential to find alternatives or accommodations for children who are absent from school or unable to participate while at school due to migraines. Future studies should continue investigating how technology, including videoconferencing software, such as Zoom, can allow children to participate in the educational process at school even if they are at home [38].

### 4.4. Effects of the COVID-19 Pandemic on Children with Migraine

Our study, conducted during the COVID-19 epidemic, explored the influence of this unique period on pediatric migraine headaches. We found no worsening of headaches during the pandemic among children with migraine. Moreover, our second hypothesis—positing that children with migraine who contracted COVID-19 would have the worst outcomes—was not supported. Interestingly, the only significant difference between children with migraine who did and those who did not contract COVID-19 was in was the monitoring scale of the BRIEF, with monitoring being worse in children who did not contract COVID-19. A similar pattern was seen when we measured quality of life: among the participants who contracted COVID-19, quality of life was lower only among the control group and not among the migraine patients. These findings suggest a complex interplay among migraine, COVID-19, and neurocognitive functioning. The worse monitoring scores in children with migraine who did not contract COVID-19, as measured by the BRIEF scale, may reflect pre-existing differences in executive functioning. It is possible that children with migraine are more prone to cognitive challenges, including difficulties with self-regulation and attentional control, regardless of COVID-19 status. Additionally, the stress and lifestyle disruptions caused by the pandemic, such as school closures, social isolation, and altered daily routines, may have disproportionately affected children with migraine, leading to worsening cognitive monitoring in those who did not experience the infection itself.

Regarding quality of life, the finding that it declined only in the control group (non-migraine participants) after COVID-19, but not in those with migraine, could indicate a resilience factor in migraine patients. Children with chronic illnesses, like migraine, may have already developed coping mechanisms to manage health-related challenges, making them less susceptible to further declines in quality of life post-COVID-19. Alternatively, migraine itself may overshadow the impact of COVID-19 on quality of life, meaning that additional stressors related to COVID-19 did not significantly alter their already affected baseline well-being. Further research is needed to explore these potential explanations.

Another interesting finding is that among all groups, children who did not contract COVID-19 had lower EF and higher anxiety rates compared to those who did develop COVID-19 symptoms. This finding may suggest that, among children, fear and worry about COVID-19 typically had a more deleterious effect than contracting the disease itself.

The influence of the COVID-19 pandemic on headache is a matter of debate. Al-Hashel [39] reported that the COVID-19 pandemic had a predominantly negative effect on adults with migraine, highlighting the need to identify risk factors associated with poorer outcomes. Reyes-Alvarez et al. [10] investigated 243 migraine patients who responded to questions covering quarantine circumstances, alterations in working conditions, symptoms of anxiety and depression, and fear of COVID-19. About half the migraine patients they surveyed reported a deterioration in symptoms, while about a third experienced no change in their migraine symptoms.

According to Caronna et al. [7], approximately 50% of patients with headaches attributed to SARS-CoV-2 infection perceived the acute headache phase to be more severe than their typical migraine. Additionally, patients who experienced headaches during the acute phase of the infection showed higher rates of migraine exacerbation compared to those who did not. Fernández-de-las-Peñas et al. [40] found that six months after contracting COVID-19, approximately 60% of patients with a pre-existing history of migraine reported an increase in headache frequency, and around 20% reported an increase in headache intensity. Membrilla et al. [41] observed that patients with a history of migraine tend to experience longer and more intense headaches in the context of COVID-19, with these symptoms often manifesting earlier compared to individuals without a history of migraines. However, some studies investigating the presence of headaches in individuals with COVID-19 fail to clearly distinguish between those who had pre-existing primary headache disorders and those who did not, leading to uncertainty when assessing their connection [41].

It is well-known that lifestyle and behavioral factors can influence the trajectory of migraine attacks. The COVID-19 pandemic introduced unprecedented circumstances, including the implementation of lockdown measures, which significantly impacted people’s behaviors. A large study examining the effects of restrictions during the pandemic [9] found that brief periods of lockdown had the potential to improve certain measures related to migraines and overall wellbeing—perhaps a function of factors such as increased ability to work from home, reduced demands from social activities, and greater freedom to organize one’s own time. Likewise, Delussi et al. [42] found that individuals with migraine experienced an improvement in their headaches and reduced their use of medication during lockdown periods, particularly among those with lower depressive symptom scores. Apetti et al. [8] reported a similar trend, observing that primary headache disorders generally improved during lockdown, even among pediatric patients. The researchers attributed these positive outcomes to lifestyle modifications, particularly reductions in school-related stress.

Considering the effects of COVID restrictions, Caronna et al. [7] reported varying results. While some of the patients they studied experienced improvement during lockdown, others found no significant difference or even a worsening of their usual headaches, accompanied by an increase in medication intake. These negative effects were often associated with sleep disturbances, anxiety, and depression.

### 4.5. Limitations

The research findings are based on a convenience sample with a small number of participants in a single center. Many confounding factors could have been involved during the different COVID-19 waves. Additionally, the groups were not completely matched in terms of socio-demographic measures. Finally, we did not examine other parameters that could affect quality of life and executive functions, such as anxiety, depression, stress, and behavioral disorders, which are known to be common in patients with migraines.

## 5. Conclusions

In our study, children with migraine exhibited higher anxiety and lower executive functions than healthy controls. Their reduced EF correlated with lower quality of life and with the intensity of the headache attacks. The COVID-19 pandemic did not have a clear worsening effect on migraine patients, as it had on the control group. Further research on pediatric migraine and its relationship with different aspects of the COVID-19 pandemic period is warranted.

## Figures and Tables

**Table 1:** Participants’ health and socio-demographic data and characteristics related to the COVID-19 pandemic.

		Children with Migraine (N = 33)	Healthy Controls (N = 51)		
Age (years)	Range	9–17.5	10–16	T	*p*
	Mean ± SD	13.30 ± 2.93	12.51 ± 2.08	1.63	0.11
	N (%)			χ^2^	*p*
Gender				0.9	0.76
	Female	16 (48.5%)	23 (45.1%)		
	Male	17 (51.5%)	28 (54.9%)		
ADHD		9 (28.1%)	11 (22%)		
Parents’ education					
Mother				4.61	0.11
	High school	6 (18.2%)	3 (6%)		
	Vocational school	5 (15.2%)	4 (8%)		
	Academic degree	22 (66.8%)	43 (86%)		
Father				1.44	0.49
	High school	4 (12.5%)	8 (16.3%)		
	Vocational school	7 (21.9%)	6 (12.2%)		
	Academic degree	21 (65.6%)	35 (71.5%)		
Household income				7.71	0.02
	Below average	10 (33.3%)	5 (10.2%)		
	Average	11 (36.7%)	29 (59.2%)		
	Above average	9 (30%)	15 (30.6%)		
Contracted COVID-19 (defined as developing symptoms)		13 (39.4%)	12 (24%)	2.238	0.13
COVID-19 era affected child’s emotional state		11 (34.4%)	25 (51%)	2.172	0.14
Child maintained a daily routine		22 (40.7%)	32 (59.3%)	0.418	0.52
COVID-19 era affected child’s academic performance		16 (39%)	25 (61%)	0.630	0.73

ADHD = attention deficit hyperactivity disorder; SD = standard deviation.

**Table 2:** Comparing EF (BRIEF scores) between children with migraine and healthy controls.

	Children with Migraine (N = 30)		Healthy Controls (N = 39)			
BRIEF Scores	Mean ± SD	Range	Mean ± SD	Range	F(1,65)	*p*
Inhibition	48.96 ± 9.49	40–72	48.56 ± 11.13	36–81	1.509	0.224
Shifting	55.76 ± 12.94	38–88	51.74 ± 10.99	38–75	0.237	0.628
Emotional control	55.80 ± 11.55	36–77	47.07 ± 10.32	35–72	2.837	0.097
Initiation	48.80 ± 8.32	36–65	50.69 ± 10.70	35–77	5.263	0.025
Working memory	52.66 ± 12.67	38–84	48.76 ± 11.97	36–84	0.277	0.600
Planning and organization	50.23 ± 12.20	38–82	50.53 ± 9.51	35–74	0.838	0.363
Organization of material	51.46 ± 11.00	34–71	47.15 ± 11.71	33–71	0.858	0.358
Monitoring	49.36 ± 10.09	36–75	45.79 ± 12.41	28–84	0.009	0.925
BRI	45.16 ± 11.25	39–80	48.58 ± 11.16	34–78	0.247	0.621
MI	51.43 ± 13.08	38–99	50.23 ± 12.62	31–86	3.107	0.083
GEC	52.16 ± 10.35	38–75	48.43 ± 11.19	32–75	0.036	0.851

BRI = behavioral regulation index, MI = metacognition index, GEC = global executive composition, SD = standard deviation, BRIEF = Behavior Rating Inventory of Executive Function.

**Table 3:** Correlations between executive functions (BRIEF) and social participation (CASP) among children with migraine.

	CASP				
	Home	Community	School	Community Living	Total
BRIEF scores (N = 20)					
Inhibition	NS	NS	NS	NS	NS
Shifting	NS	r = −0.547 * p = 0.012	NS	r = −0.539 * p = 0.014	r = −0.550 * p = 0.012
Emotional control	NS	NS	NS	r = −0.526 * p = 0.017	r = −0.537 * p = 0.015
Initiation	NS	NS	NS	NS	NS
Working memory	NS	NS	NS	NS	NS
Planning and organization	NS	NS	NS	NS	NS
Organization of material	NS	NS	NS	NS	NS
Monitoring	NS	NS	NS	r = −0.740 ** p = 0.000	r = −0.716 ** p = 0.000
BRI	NS	r = −0.463 * p = 0.040	NS	r = −0.472 * p = 0.036	r = 0.514 * p = 0.020
MI	NS	NS	NS	NS	NS
GEC	NS	NS	NS	r = −0.456 * p = 0.043	r = −0.465 * p = 0.039
PedsQL scores (N = 23)					
Emotional	NS	r = 0.469 * p = 0.024	NS	r = 0.461 * p = 0.027	r = 0.552 ** p = 0.006
Social	NS	NS	NS	r = 0.603 ** p = 0.002	r = 0.585 ** p = 0.003
School	NS	NS	NS	NS	NS
Psycho-social	NS	NS	NS	r = 0.518 * p = 0.011	r = 0.546 ** p = 0.007
Total	NS	NS	NS	r = 0.477 * p = 0.021	r = 0.535 ** p = 0.009

NS = not significant, BRI = behavioral regulation index, MI = metacognition index, GEC = global executive composition, BRIEF = Behavior Rating Inventory of Executive Function, CASP = Child and Adolescent Scale of Participation, PedsQL = Pediatric Quality of Life Inventory. * Moderate effect, ** High effect.

## Data Availability

The data presented in this study are available on request from the corresponding author.
